# ctDNA clearance predicts survival in unresectable EGFR-mutant NSCLC: a meta-analysis

**DOI:** 10.3389/fonc.2026.1743159

**Published:** 2026-02-16

**Authors:** Peixian Li, Yujiao Zhang, Fangyuan Qin, Rui Li

**Affiliations:** 1 Department of Oncology, Heilongjiang Beidahuang Group General Hospital, Harbin, Heilongjiang, China; 2 Department of Respiratory and Critical Care Medicine, Heilongjiang Beidahuang Group General Hospital, Harbin, Heilongjiang, China

**Keywords:** ctDNA clearance, EGFR mutations, meta-analysis, non-small cell lung cancer, prognostic biomarker

## Abstract

**Background:**

Circulating tumor DNA (ctDNA) is a non-invasive biomarker for monitoring non-small cell lung cancer (NSCLC). In EGFR-mutant NSCLC, ctDNA clearance during EGFR-TKI therapy is linked to improved outcomes.

**Methods:**

A systematic review was conducted following PRISMA guidelines, with data from PubMed, EMBASE, Web of Science, and Cochrane Library up to September 2025. Eligible studies were prospective cohorts or randomized trials reporting ctDNA dynamics and hazard ratios (HRs) for progression-free survival (PFS) and overall survival (OS).

**Results:**

Four studies involving a total of 336 patients met the inclusion criteria. Pooled analysis showed ctDNA clearance was significantly associated with prolonged PFS (HR = 0.34) and OS (HR = 0.29). Sensitivity analyses confirmed the robustness, and no significant publication bias was detected.

**Conclusions:**

ctDNA clearance during EGFR-TKI treatment appears to be associated with improved survival outcomes in patients with EGFR-mutant NSCLC. These findings suggest that ctDNA dynamics may serve as a promising early prognostic biomarker, although further validation in larger prospective studies and standardization of ctDNA assays, sampling time points, and clearance definitions are required.

## 1 Introduction

Among the main causes of cancer-related death, on-small cell lung cancer (NSCLC)is still in the forefront of the world (1, 2). And the EGFR-mutant subtypes constitute a significant proportion of NSCLC case (3, 4). Among patients with unresectable NSCLC carrying EGFR mutations, the use of epidermal growth factor receptor tyrosine kinase inhibitors (EGFR-TKIs), including gefitinib, lelotinib, and osimertinib, has especially improved survival results (5, 6). However, most patients eventually develop acquired resistance (7), commonly driven by secondary mutations such as T790M (3, 8). Given that standard imaging techniques provide only periodic assessments of therapeutic response, the development of real-time biomarkers to inform treatment decisions is urgently needed (9).

Circulating tumor DNA (ctDNA), which consists of plasma DNA fragments originating from tumor cells, has emerged as a non-invasive method for tracking therapeutic response and resistance (10–12). In EGFR-mutant NSCLC, ctDNA profiling allows dynamic tracking of tumor mutations and burden. Recent studies indicate that the clearance of ctDNA, detectable soon after the onset of EGFR-TKI therapy, is linked to improved progression-free survival (PFS) and overall survival (OS) (5, 13, 14). Nonetheless, the results of several investigations are heterogeneous; this could be because of variations in ctDNA measurement techniques, which are used to define the operational definitions of clearance, and because of the features of patient populations (8, 10, 15, 16).

In patients with non-surveable EGFR-mutant NSCLC receiving EGFR-TKI treatment, the purpose of this systematic review and meta-analysis was to evaluate the prognostic usefulness of ctDNA clearance. Specifically, we evaluated whether patients achieving early ctDNA clearance had significantly improved PFS and OS compared with those with persistent ctDNA, and examined the consistency of results across studies.

## 2 Methods

### 2.1 Search strategy and study selection

We conducted a systematic search of PubMed, EMBASE, Web of Science, and the Cochrane Library from their inception to September 2025, with the final search conducted on September 16, 2025. The search strategy combined keywords and MeSH terms covering EGFR-mutant NSCLC and ctDNA(e.g., (EGFR OR “epidermal growth factor receptor”) AND (NSCLC OR “non-small cell lung cancer”) AND (ctDNA OR “circulating tumor DNA”) AND (EGFR-TKI OR “tyrosine kinase inhibitor”)). The full electronic search strategies, including detailed search strings and Boolean operators for each database, are provided in **Supplementary Table S1**. There were no language limitations imposed during the search process. Two reviewers, Author A and Author B, individually screened the titles and abstracts, resolving any discrepancies through discussion or with the assistance of a third reviewer. For inclusion, whole-text publications that were found to be possibly eligible were assessed.

Eligibility standards were defined according to the PICO framework:

- Population: Patients with unresectable NSCLC harboring activating EGFR mutations;
- Exposure: ctDNA clearance during EGFR-TKI treatment, defined as undetectable or markedly reduced plasma EGFR-mutant ctDNA during the initial treatment period (typically assessed 2–4 weeks after therapy initiation; clearance threshold generally <0.1% variant allele frequency [VAF]);
- Comparison: Persistent ctDNA (detectable ctDNA at early time points);
- Outcomes: Hazard ratios (HRs) with 95% confidence intervals (CIs) were calculated for progression-free survival (PFS; primary endpoint) and overall survival (OS; secondary endpoint), comparing ctDNA clearance to its persistence;
- Study Design: Prospective cohort studies or randomized controlled trials. To reduce bias and guarantee the collection of prospective data, retrospective studies were not included. As a result, all included studies were prospective, precluding stratified analysis by study design;
- Language: English-language, peer-reviewed publications.

Studies were excluded if they did not report hazard ratios (HRs) for PFS or OS stratified by ctDNA clearance status, included cancer types or molecular alterations other than EGFR-mutant NSCLC, or enrolled patients who had received systemic chemotherapy or immunotherapy prior to ctDNA assessment. In addition, studies in which patients received concurrent systemic treatments other than EGFR-TKIs (e.g., EGFR-TKI combined with chemotherapy or immunotherapy) were excluded to minimize treatment-related confounding.

**Figure 1:**
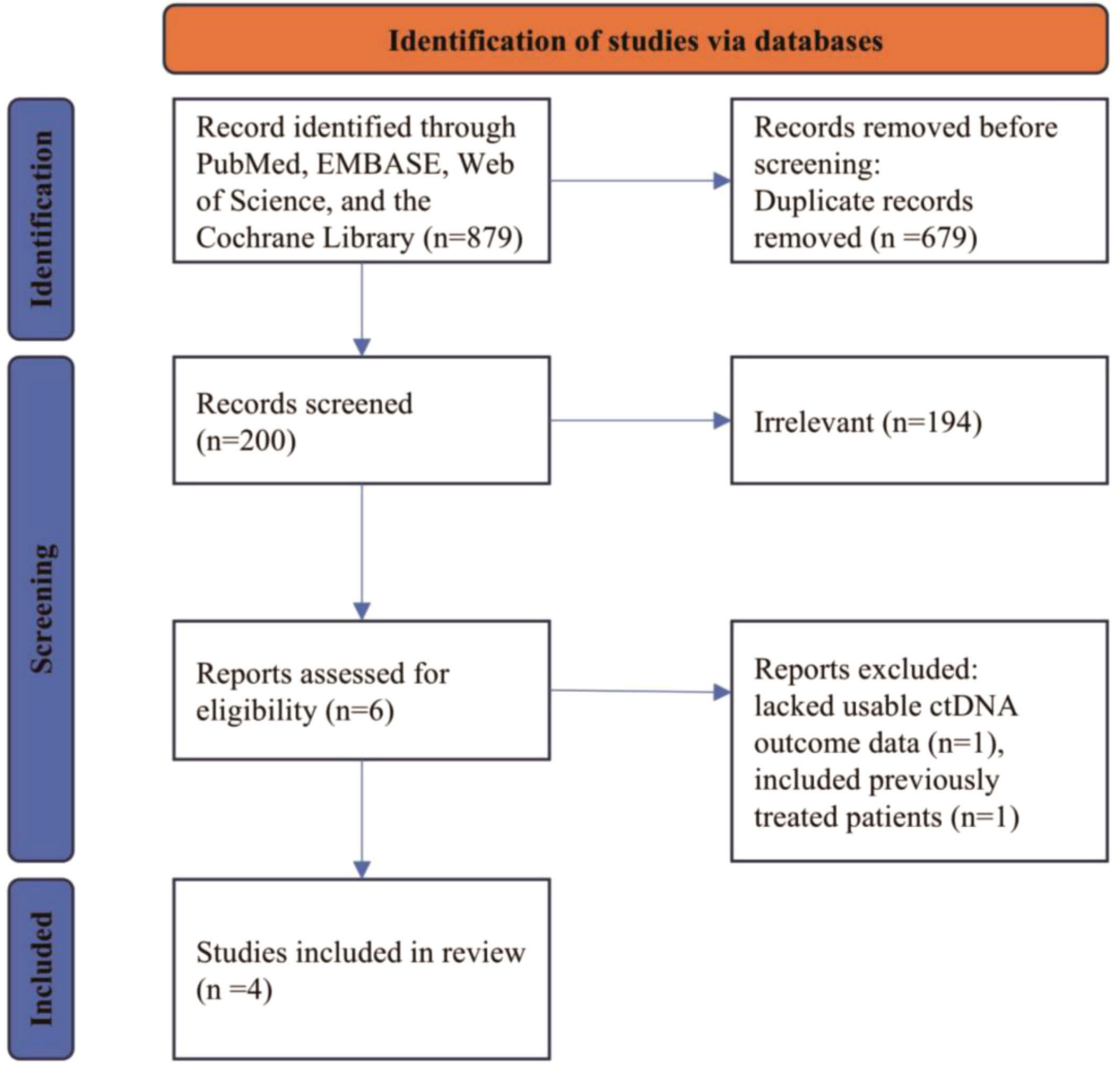
PRISMA flowchart.

The study selection process is depicted in the PRISMA flow diagram (**Figure 1**). In brief, a total of 879 records were identified. After removing 679 duplicates, 200 records underwent title/abstract screening, of which 194 were excluded as irrelevant. Six full texts were assessed, and 2 were excluded (1 lacked usable ctDNA outcome data, and 1 included previously treated patients). Ultimately, four studies satisfied all the eligibility requirements and were incorporated into the quantitative analysis.

### 2.2 Data extraction and quality assessment

Two reviewers used a standardized version of the eligible studies to independently extract data from them. The retrieved variables included the year of publication, study design, sample size, patient demographics (median age, sex distribution, disease stage), EGFR mutation subtype, EGFR-TKI regimen, follow-up length, ctDNA detection method (ddPCR or NGS), ctDNA sampling time points, definition of ctDNA clearance, and hazard ratios (HRs)with 95%confidence intervals (CIs) for PFS and OS, stratified by clearance status.

**Figure 2:**
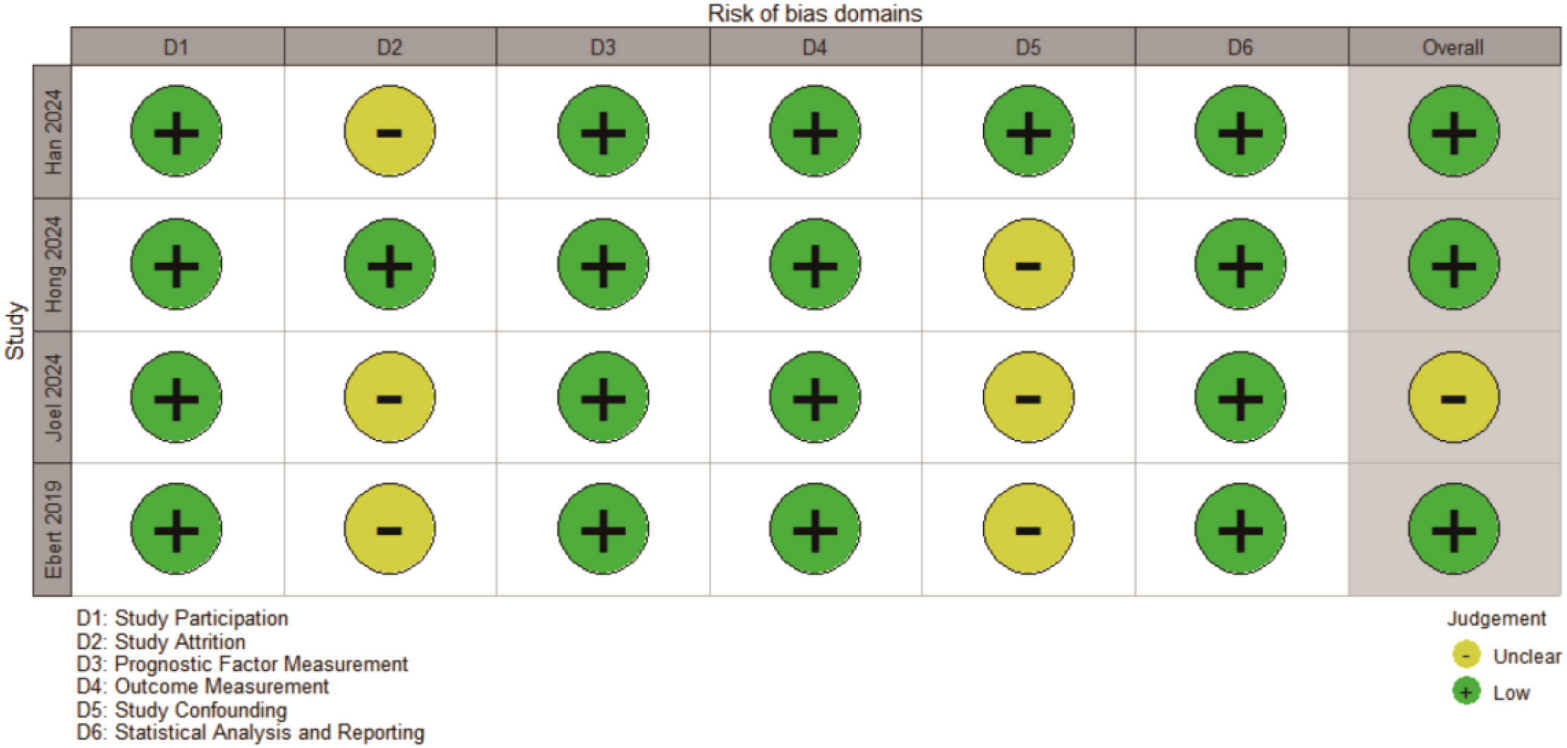
Risk of bias assessment.

In particular, the QUIPS (Quality in prognosis studies) tool was used to evaluate the risk of bias and quality of the study, which were created especially for prognostic factor studies. Participants for the study were evaluated by the QUIPS domains, which included the following: measurement of outcome, measurement of result, control of confounding, and statistical analysis/reporting (ctDNA). Each domain was assessed for risk of bias as low, moderate, or high (**Figure 2**). Any differences in data extraction or quality assessment were addressed through discussion, with a third reviewer contacted when necessary.

### 2.3 Statistical analysis

Using the software version 4.1.0, all of the meta-analytical procedures were performed. The main effect metric was the HR for PFS and OS, contrasting patients with ctDNA clearance against those with persistent ctDNA. To address variability among studies, pooled log-HRs were derived using a DerSimonian–Laird random-effects approach. Pooled HRs with 95%CIs were reported, along with estimates of between-study variance (τ ²). In addition, 95%prediction intervals were computed in order to determine the possible range of actual impacts from subsequent comparable research. The effect estimates were adjusted by applying the inverse variance method. Heterogeneity was assessed using both the I² statistic and Cochran’s Q test. I values were regarded as low, moderate, high, and high levels of heterogeneity, respectively; I values were 25%, 50%, and 75%. EGFR-TKI (first/second *vs*. third) generation (pre-specified subgroup analyses) and the ctDNA detection method were included in the pre-established subgroup analyses. As all included studies were prospective in design, subgroup analysis by study type was not feasible. Sensitivity analyses were carried out by consecutively excluding each study in order to evaluate the effect of individual studies on the total results.

Using funnel plots, the potential publication bias for both PFS and OS was qualitatively evaluated. Formal statistical tests for asymmetry (e.g., Begg’s or Egger’s test) were not performed, as the small number of included studies (k = 4) would yield insufficient power. Statistical tests were conducted using two-sided methods, with a p-value of less than 0.05 deemed statistically significant when appropriate.

## 3 Results

### 3.1 Study characteristics

A total of four studies, published between 2019 and 2024 and comprising several hundred patients, were included in this analysis. All studies enrolled patients with advanced (stage III/IV) EGFR-mutant non–small cell lung cancer (NSCLC) receiving EGFR tyrosine kinase inhibitor (EGFR-TKI) therapy. One study was a randomized controlled trial, and three were prospective cohort studies. All patients harbored sensitizing EGFR mutations, and none had received chemotherapy or immunotherapy prior to ctDNA sampling.

Baseline plasma samples were positive for EGFR-mutant ctDNA in all included studies. Early ctDNA assessment was typically performed 2–4 weeks after initiation of EGFR-TKI therapy, with minor variations across studies. Definitions of ctDNA “clearance” varied: some studies defined clearance as complete disappearance of mutant ctDNA, whereas others applied a predefined threshold, generally a variant allele frequency (VAF) of <0.1%. Accordingly, ctDNA clearance was classified based on the criteria adopted in each individual study.

Most studies detected ctDNA using droplet digital PCR (ddPCR), with one study employing a next-generation sequencing (NGS)–based panel. EGFR-TKI regimens included first-generation inhibitors (gefitinib, erlotinib), the second-generation inhibitor afatinib, and the third-generation inhibitor osimertinib, with some studies pooling patients treated with different TKI generations.

Risk of bias was assessed using the Quality In Prognosis Studies (QUIPS) tool. Overall, the included studies demonstrated low to moderate risk of bias across most domains. The most frequent sources of potential bias were related to prognostic factor measurement and confounding, whereas outcome measurement and statistical reporting were generally at low risk (**Figure 2**). Most studies adjusted for baseline patient characteristics using multivariable analyses or design-based approaches to mitigate confounding. Detailed study and patient characteristics are summarized in **Table 1**.

**Table 1:** Detailed study and patient characteristics.

Study	Country	N	Stage	EGFR-TKI regimen	ctDNA method	Clearance definition	Sampling time	Follow-up (months)	HRs reported
Han 2024	Korea	74	III–IV	Lazertinib	ddPCR	Undetectable	2–4 weeks	18 (median)	PFS, OS (adjusted)
Hong 2024	China	98	IIIB–IV	Icotinib	ddPCR	<0.1% VAF	4 weeks	15 (median)	PFS (adjusted)
Joel 2024	India	66	IIIB–IV	Gefitinib/Erlotinib/Osimertinib	NGS	Undetectable	2 weeks	20 (median)	PFS, OS (adjusted)
Ebert 2019	Denmark	98	Advanced	Erlotinib	ddPCR	Undetectable	2–4 weeks	24 (median)	PFS, OS (unadjusted)

### 3.2 Meta-analysis of PFS

**Figure 3:**
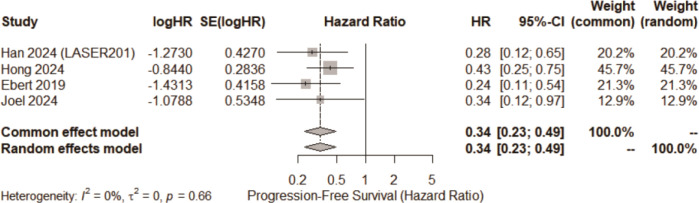
The forest plot of pooled HRs for PFS.

All four studies reported HRs for PFS according to ctDNA clearance status. The pooled analysis revealed a significant relationship between clearance and extended PFS (pooled HR = 0.34; 95%CI 0.23 – 0.49). This indicates a 66% reduction in the risk of disease progression among patients achieving early ctDNA clearance. Statistical heterogeneity was negligible (I² = 0%; τ² ≈ 0), suggesting high consistency across studies. Although the 95% prediction interval was relatively wide due to the small number of studies, it remained clearly below 1, further reinforcing the robustness of the association (**Figure 3**).

### 3.3 Meta-analysis of OS

**Figure 4:**
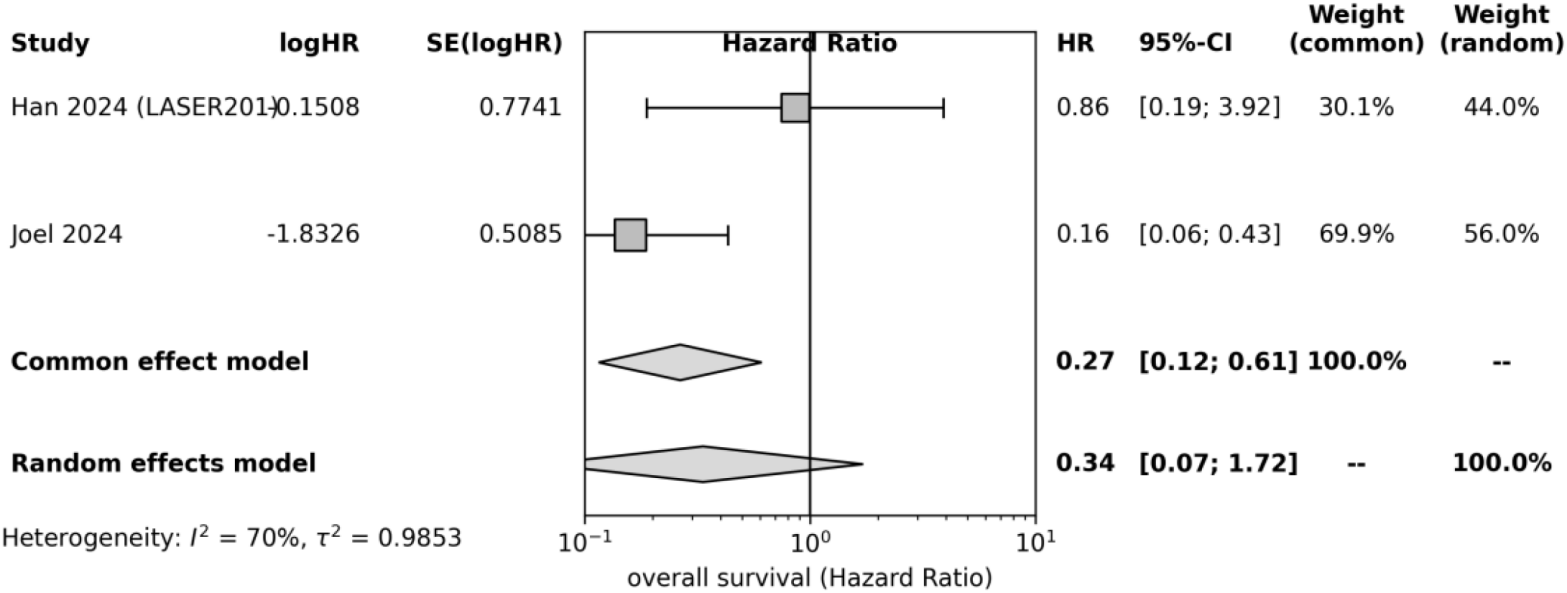
Forest plot of overall survival (OS) according to ctDNA clearance status in patients with unresectable EGFR-mutant NSCLC treated with EGFR-TKIs. Only studies reporting extractable OS hazard ratios were included in this analysis.

For OS, there was a strong survival advantage in the early ctDNA clearance process as well. The pooled HR was 0.29 (95% CI: 0.11–0.75), corresponding to an approximately 71% reduction in mortality risk. Heterogeneity was low (I² = 39%). Although the confidence interval was wider, reflecting fewer events and patients included in OS analyses, the result remained statistically significant. Prediction intervals were also relatively broad given limited data, yet all point estimates were <1 (**Figure 4**).

### 3.4 Sensitivity analyses

When one study was excluded at a time, sensitivity analyses revealed that no single study material changed the pooled estimates for OS or PFS; all HRs that were recalculated stayed constant in both direction and magnitude. According to this, none of the outliers in our research were used to determine the general conclusions.

### 3.5 Publication bias

**Figure 5:**
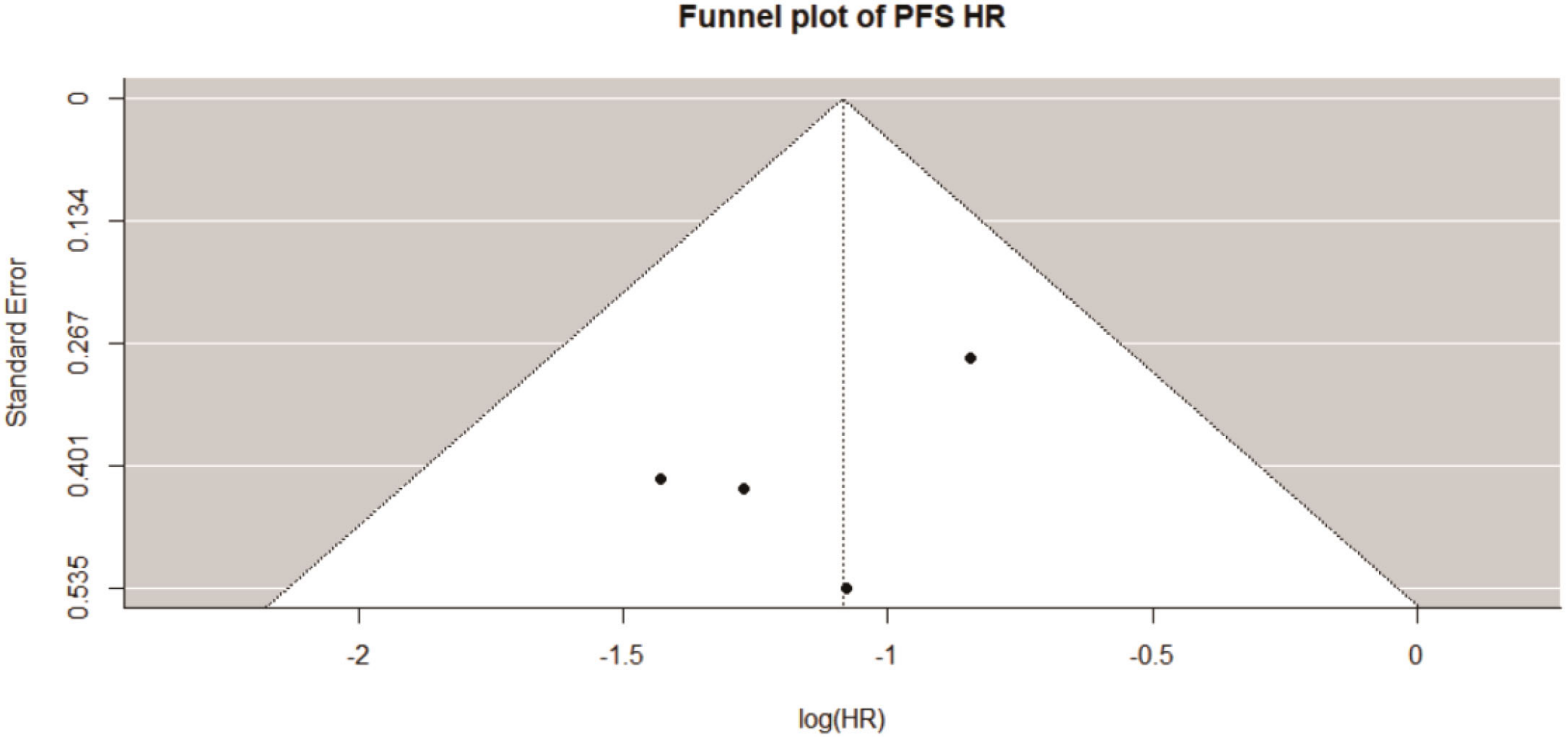
Funnel plots.

The statistical tests for publication bias lacked adequate power due to the inclusion of only four studies. There was no evident asymmetry (**Figure 5**) in the PFS and OS funnel plots, but the assessment was only qualitative. Although we cannot discount the existence of unpublished negative studies, the consistency and strength of the observed associations indicate that significant publication bias is improbable to entirely explain our findings.

### 3.6 Limitations

This meta-analysis has several limitations:

1. Only four studies were included, with limited total sample size and statistical power, particularly affecting OS estimates and subgroup analyses.
2. Despite low statistical heterogeneity, clinical heterogeneity existed in terms of EGFR-TKI regimens, ctDNA detection platforms, sampling times (approximately 2–4 weeks), and clearance definitions (undetectable *vs*. threshold-based). We attempted to address this through standardized analytic frameworks and subgroup analyses, but residual heterogeneity may remain.
3. Restriction to prospective studies enhanced internal validity but limited applicability, as retrospective data (excluded from this review) might yield different results.
4. With only four studies available, funnel plots and bias assessments were inherently underpowered; thus, publication bias cannot be fully excluded.

## 4 Discussion

This meta-analysis demonstrates that ctDNA clearance following initiation of EGFR-TKI therapy significantly predicts improved clinical outcomes in advanced NSCLC. The pooled hazard ratios for both PFS and OS (approximately 0.3) indicate a substantial effect size, suggesting that early ctDNA clearance is strongly associated with prolonged survival. This finding aligns with the biological hypothesis that a rapid decline in tumor-derived DNA reflects effective tumor killing and is closely linked to treatment sensitivity.

Compared with conventional imaging, ctDNA provides a real-time molecular snapshot of treatment response (10, 17). In clinical practice, ctDNA assessment at the first treatment milestone (e.g., 2–4 weeks after initiation) may give therapeutic feedback prior to radiographic changes, permitting early intervention for non-responders (such as treatment modification) (5). The clinical value of including monitoring tactics in EGFR-mutant NSCLC is supported by our data.

To facilitate broader clinical application and comparability across studies, future research should aim to standardize key methodological parameters. Based on the current evidence, early ctDNA assessment within 2–4 weeks after initiation of EGFR-TKI therapy appears to be a pragmatic time window, while variant allele frequency (VAF) thresholds below 0.1% may serve as a reasonable preliminary definition of molecular clearance. These parameters, however, require prospective validation across different platforms and clinical settings.

From a clinical perspective, ctDNA clearance has the potential to complement radiographic response assessment. Rather than replacing conventional imaging-based criteria such as RECIST, early ctDNA dynamics could be integrated as an adjunct tool to identify patients at high risk of early progression despite radiographic stability, thereby informing closer monitoring or early treatment adaptation.

Beyond the pooled effect estimates, this study makes a distinct contribution by synthesizing available evidence on the prognostic relevance of ctDNA clearance in a clearly defined clinical context. To our knowledge, this is the first systematic review and meta-analysis to quantitatively integrate survival outcomes associated with ctDNA clearance specifically in patients with unresectable EGFR-mutant NSCLC treated with EGFR-TKIs. By focusing on dynamic molecular response rather than baseline ctDNA status, our findings consolidate emerging evidence supporting ctDNA kinetics as an early prognostic indicator in this molecularly selected population.

Several limitations of this study should be acknowledged. First, the number of included studies was limited, which may restrict statistical power and generalizability. Due to the small number of included studies, the risk of publication bias cannot be reliably assessed, and funnel plots should be interpreted descriptively. Consequently, subgroup analyses according to TKI generation or ctDNA detection method are likely underpowered and should be interpreted with caution, serving primarily as exploratory assessments rather than definitive comparisons.

Second, important methodological heterogeneity warrants consideration. Clinical heterogeneity existed in ctDNA clearance definitions (undetectable versus threshold-based), sampling time points, and analytical platforms, which may influence pooled effect estimates despite low statistical heterogeneity. For instance, ddPCR assays can detect extremely low variant allele frequencies (VAFs; approximately 0.01–0.1%), whereas some next-generation sequencing (NGS) platforms have higher limits of detection. Although our pooled analyses demonstrated consistent survival associations across platforms, such analytical differences may influence clearance rates and effect estimates. In addition, the timing of ctDNA assessment and thresholds used to define clearance varied across studies. While clearance is commonly conceptualized within 2–4 weeks of treatment initiation and at VAF levels below 0.1%, these criteria are not universally standardized, limiting comparability across studies and current clinical applicability.

Third, statistical considerations should be noted. All pooled analyses were conducted using DerSimonian–Laird random-effects models, with heterogeneity assessed by τ² and I² statistics. Although low observed heterogeneity increases confidence in the consistency of the findings, the small number of studies limits the power to detect between-study variability. Accordingly, prediction intervals were reported to reflect uncertainty when extrapolating results to new clinical settings.

Fourth, with respect to study design and evidence quality, inclusion was restricted to prospective studies to minimize bias, such as preferential enrollment of responders. Risk-of-bias assessment using the QUIPS tool suggested overall moderate-to-good methodological quality across studies; however, limited sample sizes constrained the precision of effect estimates. Sensitivity analyses confirmed the robustness of the results, indicating that no single study disproportionately influenced the pooled outcomes.

Finally, the generalizability of ctDNA clearance as a prognostic biomarker may be limited in tumors with intrinsically low DNA shedding, such as indolent malignancies, minimal residual disease, or tumors confined to anatomical compartments with restricted DNA release. In these settings, undetectable ctDNA may reflect biological low shedding rather than true molecular response. Moreover, ctDNA shedding varies by metastatic site, with lesions in the brain, bone, or other sanctuary sites often associated with lower detectable ctDNA levels, potentially leading to underestimation of residual disease burden. Different ctDNA detection platforms, including ddPCR and NGS, also exhibit variable analytical sensitivity and limits of detection, which may further influence clearance classification. Taken together, these factors underscore the importance of interpreting ctDNA clearance in conjunction with radiographic and clinical assessment, rather than as a standalone indicator. Ultimately, although these data are persuasive, validation in bigger prospective trials is required. Future studies should establish standardized definitions of ctDNA clearance and assess whether ctDNA-guided treatment adaptation improves outcomes. For example, randomized trials could allocate patients without clearance to either treatment modification or continuation of the original regimen to directly test clinical benefit.

## 5 Conclusions

In conclusion, this systematic review and meta-analysis suggest that ctDNA clearance during EGFR-TKI treatment appears to be associated with improved survival outcomes in patients with unresectable EGFR-mutant NSCLC. Patients achieving ctDNA clearance tended to experience longer progression-free survival and overall survival. While these findings highlight the potential clinical value of ctDNA dynamics as an early prognostic biomarker, further validation in larger, prospective studies is warranted. Future research should focus on standardizing ctDNA detection methods, including sampling time points and clearance definitions, and on determining whether ctDNA-guided treatment strategies can translate into improved patient outcomes.

## Data Availability

The raw data supporting the conclusions of this article will be made available by the authors, without undue reservation.
