# Supplementary material for: ctDNA clearance predicts survival in unresectable EGFR-mutant NSCLC: a meta-analysis
Source: Front Oncol. 2026 Feb 16;16:1743159. doi: 10.3389/fonc.2026.1743159 (PMC12950585; doi:10.3389/fonc.2026.1743159)
Supplement: Supplementary file 1 [file Table1.docx]

**Table S1.** Search strategies used in different databases

| **Database** | **Search Strategy** |
| --- | --- |
| **PubMed** | ((“Lung Neoplasms”[Mesh] OR “Carcinoma, Non-Small-Cell Lung”[Mesh] OR “Carcinoma, Small Cell”[Mesh] OR “lung cancer”[tiab] OR “lung neoplasm”[tiab] OR “lung tumor”[tiab] OR “lung tumour”[tiab] OR “pulmonary cancer”[tiab] OR “pulmonary neoplasm”[tiab] OR “pulmonary tumor”[tiab] OR “pulmonary tumour”[tiab] OR “bronchogenic carcinoma”[tiab] OR “NSCLC”[tiab] OR “SCLC”[tiab] OR “Non-Small Cell Lung Cancer”[tiab] OR “Small Cell Lung Cancer”[tiab]) AND (“Circulating Tumor DNA”[Mesh] OR “Cell-Free Nucleic Acids”[Mesh] OR “circulating tumor DNA”[tiab] OR “circulating tumour DNA”[tiab] OR “ctDNA”[tiab] OR “cell-free DNA”[tiab] OR “cell free DNA”[tiab] OR “cfDNA”[tiab] OR “plasma DNA”[tiab]) AND (“unresectable”[tiab] OR “inoperable”[tiab] OR “advanced”[tiab] OR “stage III”[tiab] OR “stage IV”[tiab]) AND (“targeted therapy”[tiab] OR “Molecular Targeted Therapy”[Mesh])) |
| **Web of Science** | TS=( (“lung cancer” OR “lung neoplasm*” OR “lung tumor*” OR “lung tumour*” OR “pulmonary cancer” OR “pulmonary neoplasm*” OR “pulmonary tumor*” OR “pulmonary tumour*” OR “bronchogenic carcinoma” OR NSCLC OR SCLC OR “non-small cell lung cancer” OR “small cell lung cancer”) AND (“circulating tumor dna” OR “circulating tumour dna” OR ctDNA OR “cell-free dna” OR “cell free dna” OR cfDNA OR “plasma dna”) AND (unresectable OR inoperable OR advanced OR “stage III” OR “stage IV”) AND (“targeted therapy” OR “molecular targeted therapy”) ) |
| **EMBASE** | (‘lung cancer’/exp OR ‘non small cell lung cancer’/exp OR ‘small cell lung cancer’/exp OR “lung cancer”:ti,ab,kw OR “lung neoplasm*”:ti,ab,kw OR “lung tumor*”:ti,ab,kw OR “lung tumour*”:ti,ab,kw OR “pulmonary cancer”:ti,ab,kw OR “pulmonary neoplasm*”:ti,ab,kw OR “pulmonary tumor*”:ti,ab,kw OR “pulmonary tumour*”:ti,ab,kw OR “bronchogenic carcinoma”:ti,ab,kw OR nsclc:ti,ab,kw OR sclc:ti,ab,kw OR “non-small cell lung cancer”:ti,ab,kw OR “small cell lung cancer”:ti,ab,kw) AND (‘circulating tumor dna’/exp OR ‘cell free dna’/exp OR “circulating tumor dna”:ti,ab,kw OR “circulating tumour dna”:ti,ab,kw OR ctdna:ti,ab,kw OR “cell-free dna”:ti,ab,kw OR “cell free dna”:ti,ab,kw OR cfdna:ti,ab,kw OR “plasma dna”:ti,ab,kw) AND (unresectable:ti,ab,kw OR inoperable:ti,ab,kw OR advanced:ti,ab,kw OR “stage iii”:ti,ab,kw OR “stage iv”:ti,ab,kw) AND (‘targeted therapy’/exp OR “targeted therapy”:ti,ab,kw OR “molecular targeted therapy”:ti,ab,kw) |
| **Cochrane Library** | #1 MeSH descriptor: [Lung Neoplasms] explode all trees#2 MeSH descriptor: [Carcinoma, Non-Small-Cell Lung] explode all trees#3 MeSH descriptor: [Carcinoma, Small Cell] explode all trees#4 (“lung cancer” OR “lung neoplasm*” OR “lung tumor*” OR “lung tumour*” OR “pulmonary cancer” OR “pulmonary neoplasm*” OR “pulmonary tumor*” OR “pulmonary tumour*” OR “bronchogenic carcinoma” OR NSCLC OR SCLC OR “non-small cell lung cancer” OR “small cell lung cancer”):ti,ab,kw#5 #1 OR #2 OR #3 OR #4#6 MeSH descriptor: [Circulating Tumor DNA] explode all trees#7 MeSH descriptor: [Cell-Free Nucleic Acids] explode all trees#8 (“circulating tumor dna” OR “circulating tumour dna” OR ctDNA OR “cell-free dna” OR “cell free dna” OR cfDNA OR “plasma dna”):ti,ab,kw#9 #6 OR #7 OR #8#10 (unresectable OR inoperable OR advanced OR “stage III” OR “stage IV”):ti,ab,kw#11 MeSH descriptor: [Molecular Targeted Therapy] explode all trees#12 (“targeted therapy” OR “molecular targeted therapy”):ti,ab,kw OR #11#13 #5 AND #9 AND #10 AND #12 |
